# Supplementary figures and images for: Unmet vaccination need among children under the age of five attending the paediatric emergency department: a cross-sectional study in a large UK district general hospital
Source: BMJ Open. 2023 Jun 26;13(6):e072053. doi: 10.1136/bmjopen-2023-072053 (PMC10410832; doi:10.1136/bmjopen-2023-072053)

Supplementary File 1 – Age distribution of children in the study

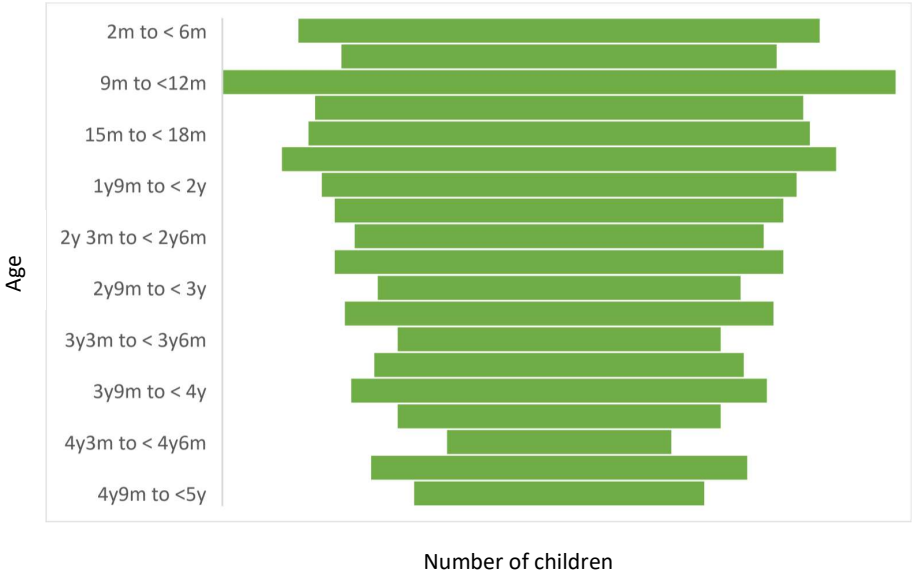

Supplement: Supplementary data [file bmjopen-2023-072053supp001.pdf]
