# Supplementary material for: Unmet vaccination need among children under the age of five attending the paediatric emergency department: a cross-sectional study in a large UK district general hospital
Source: BMJ Open. 2023 Jun 26;13(6):e072053. doi: 10.1136/bmjopen-2023-072053 (PMC10410832; doi:10.1136/bmjopen-2023-072053)
Supplement: Supplementary data [file bmjopen-2023-072053supp002.pdf]

Supplementary file 2 – Data to support figure 1

Age band	Total number of children attending	Total number of children attending with vaccination data available	Children 'up-to-date' with age-appropriate tetanus and/or MMR vaccination at attendance (n (%))	Children 'up-to-date' with age-appropriate tetanus at attendance (n (%))	Children 'up-to-date with MMR 1 at attendance (n (%))	Up to date with MMR 2 at attendance
2m to < 6m	91	79	69 (87.3)	69 (87.3)		
6m to < 9m	74	66	58 (87.9)	58 (87.9)		
9m to <12m	113	102	98 (96.1)	98 (96.1)		
12m to < 15m	80	74	52 (70.3)	69 (93.2)	48 (64.9)	
15m to < 18m	86	76	61 (80.3)	70 (92.1)	61 (80.3)	
18m to < 1y9m	90	84	72 (85.7)	78 (92.9)	75 (89.3)	
1y9m to < 2y	77	72	61 (84.7)	66 (91.7)	62 (86.1)	
2y to < 2y3m	72	68	61 (89.7)	66 (97.1)	62 (91.2)	
2y 3m to < 2y6m	68	62	56 (90.3)	60 (96.8)	56 (90.3)	
2y 6m to < 2y9m	71	68	64 (94.1)	67 (98.5)	64 (94.1)	
2y9m to < 3y	62	55	48 (87.3)	50 (90.9)	48 (87.3)	
3y to < 3y3m	70	65	35 (53.8)	63 (96.9)	35 (53.8)	
3y3m to < 3y6m	53	49	7 (19.4)	33 (67.3)	20 (55.6)	
3y4m to <3y6m	*39*	*36*	*2 (5.6)**	*20 (55.6)*	*9 (23.1)*	*2 (5.6)*
3y6m to < 3y9m	60	56	7 (12.5)	34 (60.7)	19 (33.9)	7 (12.5)
3y9m to < 4y	69	63	18 (28.6)	49 (77.8)	27 (42.9)	19 (30.2)
4y to < 4y3m	53	49	7 (14.3)	29 (59.2)	14 (28.6)	7 (14.3)
4y3m to < 4y6m	39	34	6 (17.6)	15 (44.1)	10 (29.4)	6 (17.6)
4y6m to < 4y9m	59	57	18 (31.6)	45 (78.9)	23 (40.4)	18 (31.6)
4y9m to <5y	50	44	9 (20.5)	31 (70.5)	16 (36.4)	9 (20.5)
TOTAL	1337	1259	807 (66.0)	1050 (85.9)	640 (65.6)	68 (20.1)

*Denominator for calculation is number of children aged 3y4m < 3yr 6m (n=36)

BMJ Publishing Group Limited (BMJ) disclaims all liability and responsibility arising from any reliance placed on this supplemental material which has been supplied by the author(s)

Supplementary file 2 – Data to support figure 1
