# Supplementary material for: Unmet vaccination need among children under the age of five attending the paediatric emergency department: a cross-sectional study in a large UK district general hospital
Source: BMJ Open. 2023 Jun 26;13(6):e072053. doi: 10.1136/bmjopen-2023-072053 (PMC10410832; doi:10.1136/bmjopen-2023-072053)
Supplement: Author's manuscript [file bmjopen-2023-072053.draft_revisions.pdf]

BMJ Open is committed to open peer review. As part of this commitment we make the peer review history of every article we publish publicly available.

When an article is published we post the peer reviewers' comments and the authors' responses online. We also post the versions of the paper that were used during peer review. These are the versions that the peer review comments apply to.

The versions of the paper that follow are the versions that were submitted during the peer review process. They are not the versions of record or the final published versions. They should not be cited or distributed as the published version of this manuscript.

BMJ Open is an open access journal and the full, final, typeset and author-corrected version of record of the manuscript is available on our site with no access controls, subscription charges or pay-per-view fees (http://bmjopen.bmj.com).

If you have any questions on BMJ Open's open peer review process please email info.bmjopen@bmj.com

BMJ Open

Unmet vaccination need amongst children under the age of five attending the Paediatric Emergency Department: a cross-sectional study in a large UK district general hospital.

Journal:	*BMJ Open*
Manuscript ID	bmjopen-2023-072053
Article Type:	Original research
Date Submitted by the Author:	20-Jan-2023

Complete List of Authors:	Isba, Rachel; Lancaster University; Manchester University NHS Foundation Trust<br>Brennan, Louise; Lancaster University; Manchester University NHS Foundation Trust<br>Egboko, Fiona; Lancaster University<br>Edge, Rhiannon; Lancaster University<br>Davies, Nigel; Lancaster University<br>Knight, Joanne; Lancaster University

Keywords:	PUBLIC HEALTH, Paediatric infectious disease & immunisation < PAEDIATRICS, Public health < INFECTIOUS DISEASES

SCHOLARONE™ Manuscripts

I, the Submitting Author has the right to grant and does grant on behalf of all authors of the Work (as defined in the below author licence), an exclusive licence and/or a non-exclusive licence for contributions from authors who are: i) UK Crown employees; ii) where BMJ has agreed a CC-BY licence shall apply, and/or iii) in accordance with the terms applicable for US Federal Government officers or employees acting as part of their official duties; on a worldwide, perpetual, irrevocable, royalty-free basis to BMJ Publishing Group Ltd ("BMJ") its licensees and where the relevant Journal is co-owned by BMJ to the co-owners of the Journal, to publish the Work in this journal and any other BMJ products and to exploit all rights, as set out in our licence.

The Submitting Author accepts and understands that any supply made under these terms is made by BMJ to the Submitting Author unless you are acting as an employee on behalf of your employer or a postgraduate student of an affiliated institution which is paying any applicable article publishing charge ("APC") for Open Access articles. Where the Submitting Author wishes to make the Work available on an Open Access basis (and intends to pay the relevant APC), the terms of reuse of such Open Access shall be governed by a Creative Commons licence – details of these licences and which Creative Commons licence will apply to this Work are set out in our licence referred to above.

Other than as permitted in any relevant BMJ Author's Self Archiving Policies, I confirm this Work has not been accepted for publication elsewhere, is not being considered for publication elsewhere and does not duplicate material already published. I confirm all authors consent to publication of this Work and authorise the granting of this licence.

For peer review only

Unmet vaccination need amongst children under the age of five attending the Paediatric Emergency Department: a cross-sectional study in a large UK district general hospital.

Isba, Rachel [1, 2] rachel.isba@lancaster.ac.uk (0000-0002-2896-4309), Professor
Brennan, Louise [1, 2, 3] louise.brennan8@nhs.net (0000-0003-0377-6720), Public Health Registrar
Egboko, Fiona [1] f.egboko@lancaster.ac.uk, Medical Student
Edge, Rhiannon [1] r.edge@lancaster.ac.uk (0000-0001-5349-783X), Lecturer
Davies, Nigel [4] n.a.davies@lancaster.ac.uk (0000-0002-4136-5300), Professor
Knight, Jo [1] jo.knight@lancaster.ac.uk (0000-0002-7148-1660), Professor

[1] Lancaster Medical School, Lancaster University, Lancaster, UK

[2] Royal Manchester Children's Hospital, Manchester University NHS Foundation Trust, Manchester, UK

[3] St Helens and Knowsley Teaching Hospitals NHS Trust, St Helens, UK

[4] School of Computing and Communication, Lancaster University, Lancaster, UK

Corresponding Author: Rachel Isba Rachel.isba@lancaster.ac.uk

For peer review only

Abstract

Objective

The objective was to estimate vaccination coverage amongst children under the age of five attending the Paediatric Emergency Department (PED) using tetanus and MMR vaccination as a proxy.

Design

A cross-sectional observational study with a single data collection point for each participant.

Setting

A single large PED in Greater Manchester, England.

Participants

Participants were children (under 5 years old) attending the PED during October 2021. Participation was "opt-out" and parents/carers were given until the end of the following month to request that their child's data be excluded.

Primary and secondary outcome measures

The primary outcome of interest was the percentage of children who were up-to-date with their routine childhood vaccinations at their time of attendance to the PED. Secondary outcome measures were the percentage of children who had received age-appropriate tetanus and MMR vaccination, and how these compared to local population data at the ages of 1, 2, and 5 years of age.

Results

One third of under-5s in this study had unmet vaccination need and were missing at least one dose of either MMR or tetanus-containing vaccine. In older age groups,

For peer review only

many were missing their tetanus boosters and only 1 in 5 of those eligible had received two doses of MMR. Those in younger age groups had vaccination coverage levels comparable to the local data, but still below the target of 95%.

Conclusions

Those children eligible for pre-school boosters (tetanus and MMR2) appear to have considerable unmet vaccination need. Whilst the pandemic has had an impact, the observation that MMR2 uptake is considerably lower than tetanus booster (when they are scheduled together) warrants further investigation. Catch-up campaigns for MMR2 should focus on this cohort of children and the PED may offer an opportunity for an intervention.

Trial registration

The study was registered as an observational/non-interventional study on the clinicaltrials.gov website with the identifier NCT04485624.

Word Count: 3468

Key words:

Public health, Paediatric infectious disease & immunisation, public health infectious diseases

For peer review only

Article Summary

Strengths and limitations of this study

- This study is timely given the pandemic's impact on routine childhood vaccination.
- A population has been identified with considerable unmet vaccination need who might benefit from an intervention not currently offered.
- A limitation is that the work was undertaken at a single centre and there were constraints with data collection and quality.
- A proxy for overall vaccination status was used, leading to a possible underestimation of unmet vaccination need.

For peer review only

Introduction

Vaccines are one of the great global health successes. Since their discovery more than 300 years ago, vaccines have saved countless millions of lives (1), reduced the incidence of dozens of diseases, and even lead to the eradication of smallpox (2). In countries with access to them, vaccines have also played a key part in the control of the current SARS-CoV-2/COVID-19 pandemic (3).

However, pre-pandemic, in the UK, uptake of routine childhood vaccination had fluctuated (4) and coverage lagged behind some of our European peers for common vaccine-preventable diseases such as measles (5).

Every year in England, millions of children and young people (CYP) attend hospital (secondary or tertiary medical care) (6). Attendance is often with relatively minor illnesses and injuries, many of which could be better managed elsewhere. However, despite numerous initiatives to redirect these CYP, hospital attendances (pre-pandemic) had increased year on year (6). The pronounced decrease in Paediatric Emergency Department (PED) attendances seen early in the pandemic (7,8) was reversed in 2021, as lockdowns and other restrictions were eased, with attendance exceeding pre-pandemic levels and a change in the some of the seasonal patterns of illnesses presenting to the PED (9).

In addition to their primary reason for presentation, CYP attending the hospital may have lower than average levels of health and wellbeing, additional unmet health need (e.g. dental health), or not be able to engage with preventive elements of routine healthcare (e.g. vaccination) for a myriad of reasons. A hospital attendance or admission might therefore offer an opportunity to intervene. A recent pilot study showed that time during a PED department attendance could be used to deliver a

For peer review only

public health intervention and that this was both feasible (within the constraints of the department) and acceptable (to all stakeholders including CYP, parents and carers, and staff in the PED) (10).

If any child or young person who had not received their age-appropriate routine vaccinations could be identified during a PED attendance, clinicians might (should it be clinically/situationally appropriate) be able to offer one or more tailored interventions to address this unmet vaccination need. The benefits of such an approach are numerous and include:

- decreasing mortality and morbidity from vaccine-preventable diseases, by ensuring
    - individual and population coverage for diseases that cannot spread person-person e.g. tetanus
    - higher levels of population coverage for non-epidemic diseases that can be spread person-person e.g. Hepatitis B
    - and herd immunity for diseases that can easily spread person-person and can cause outbreaks e.g. measles;
- a decrease in un-needed treatment in the case of individual exposure in the absence of an accurate vaccination history at the point of treatment e.g. a tetanus-prone wound in the PED;
- a reactive response to outbreaks e.g. mumps, epidemics e.g. influenza, and pandemics e.g. SARS-CoV-2;
- improving coverage of targeted vaccination programmes e.g. seasonal influenza.

For peer review only

In its 2021 Health Equity Audit of the National Immunisation Programme (11), Public Health England (now the UK Health Security Agency) stated "Equality in immunisation is an important way to address health inequalities" and reported that whilst the Immunisation Programme had achieved high coverage in the population as a whole, within sub-populations there still existed "avoidable inequalities". Whilst the reasons for these inequalities are complex, "institutional" and "policy" factors play a role (11). Addressing these factors by offering vaccination at the point of PED attendance may preferentially improve vaccination uptake amongst those experiencing avoidable inequalities.

Previous work to improve vaccination uptake via interventions delivered in secondary or tertiary care (see (12) for overview) has shown that vaccination coverage in CYP attending hospital settings is generally lower than in the general population. However, the vaccinations under investigation, location of presentation within the hospital, and way in which vaccination data were verified, varies considerably. Within the scoping review, for all due/overdue vaccinations (not a measure currently available in England), baseline coverage ranged from 44% (13,14) – 89% (15), with little difference by setting and a trend for lower coverage in older studies. For influenza, baseline coverage was lower, ranging from 25% (16) - 50.5% (17).

The overall aim of this work was to look at unmet vaccination need by answering the question: Do children (aged < 5 years) attending the PED have lower levels of vaccination coverage than their peers in the general population?

For peer review only

Methods

This was a cross-sectional observational study with a single data collection point for each child. The study was registered as an observational/non-interventional study on the clinicaltrials.gov website with the identifier NCT04485624.

Consent

Full prospective ethics approval was obtained from the North West – Greater Manchester East Ethics Committee (IRAS reference 278815, REC reference 20/NW/0423). Following an earlier part of the work, an ethics amendment was submitted and approved to move to "opt-out" consent for this part of the project (Amendment 1, substantial, non-CTIMP, approved 26 August 2021).

Prominently-displayed posters relating to the study were put up around the PED during the month of data collection, with flyers handed out in triage, and participant information sheets with opt-out consent forms available on request. Nursing staff in the PED were briefed so that they could support queries from parents/carers. Parents/carers could choose for their child to opt-out by filling in the form or contacting a member of the research team within a month of attendance.

For peer review only

Setting

Data were collected from a single, dedicated co-located PED, in Greater Manchester, in the North West of England.

Participants

Participants were children (under 5 years old) attending the PED during October 2021. Children and young people in Manchester have lower than average levels of health and wellbeing, around a third live in low-income households, and 1 in 100 of them reside in care (18).

Patient and public involvement

Reading materials were designed to be read by the parent or caregiver. Following patient involvement in a previous study (10) all reading materials were designed for a reading age of 7-9 years old, in order to be as accessible as possible.

Vaccination schedule

The routine NHS vaccination schedule (19) recommends tetanus-containing vaccines (given as multi-component vaccines) are given at 8, 12, and 16 weeks ("primary course") and at around 3 years 4 months (as part of the so-called "pre-school booster"). MMR1 is due around one year of age and MMR2 is given at 3 years 4 months, alongside the pre-school booster.

Data collection

A list of dates of birth and hospital numbers was generated for all attendees during the month of October 2021. Duplicates (where the same child may have attended more than once in the month of interest) were removed. Exact age at presentation was not available, so was calculated based on age in completed months on October 1st 2021. Any vaccination before the end of October 2021 was included when checking status. This approach was taken to ensure that the data tended to over- rather than under-estimate coverage e.g. at the lower age limits.

Data were extracted from individual Summary Care Records (SCRs) by RI and anonymised before analysis. Extraction took between 2 and 5 minutes per record (depending on the quality of network access/connectivity). The quality and presentation of the data within the SCR was also very variable and this finding is presented elsewhere (20).

For peer review only

The outcomes of interest were numbers of children aged:

- > 2 months and < 5 years who were "up to date" with all their age-appropriate tetanus-containing and MMR vaccinations at the time of PED attendance (this was used as a proxy for overall vaccination status);
- > 2 months and < 5 years who had received all their age-appropriate tetanus vaccinations at the time of PED attendance;
- > 12 months and < 5 years who had received at least one dose of MMR at the time of PED attendance;
- between 2 and < 5 years who had received MMR1 by their 2nd birthday;
- between 3 years 4 months and < 5 years who had received two doses of MMR at the time of PED attendance;
- between 3 years 4 months and < 5 years who had received MMR2 by their 5th birthday.

Sample size calculation

The MMR vaccine was chosen for the sample size calculation as this is the vaccine that has attracted the most controversy over the past decades (resulting in lower than target coverage).

At the time the sample size calculation was carried out, the most recent data for MMR2 coverage at 5 years were available for the year 2018/19 (21). In England, coverage for MMR2 was 86.4% and for Manchester, coverage for 5 year olds was 82.1%. The sample size calculation was carried out using STATA version 16 (22) and a comparison made between population prevalence (between the "PED" and "general Manchester" populations). The sample size calculation was based on the difference between the population prevalence in Manchester of 0.82 and in the study

For peer review only

PED of 0.77, with the required power at 0.8, using a two-sided test, with alpha level set at $p < 0.05$. This resulted in an estimated sample size for a one-sample proportion test (Wald z test) of 577.

Outcomes

Primary

The primary outcome of interest was the percentage of children under the age of 5 years who were "up-to-date" with their vaccinations at the time of their PED attendance, using tetanus-containing and MMR vaccination status combined as a proxy for overall vaccination. Children were coded as "up to date" with their vaccinations if they had received all tetanus and MMR vaccinations for which they were eligible (based on their age) by 31st October 2021.

Secondary

Secondary outcomes of interest were age-appropriate tetanus-containing vaccination coverage, and uptake of MMR1 and MMR2.

Statistical Analysis

Descriptive statistics were prepared for primary and secondary outcomes. We compared our data to data published for the year April 2021 - 2022 in the cover of vaccination evaluated rapidly (COVER) programme (4). The COVER programme publishes quarterly and annual vaccination coverage statistics for children aged 12 months, 24 months, and 5 years in the UK. Data from 2021-22 was chosen as they were the most recent published data.

We mirrored the methodology used for the COVER data (23) using number of eligible persons immunised as the numerator and total number of persons in the eligible population as the denominator, to obtain a comparable sample from our

For peer review only

study in the PED. We compared our data at 12 months, 24 months, and 5 years with the publicly available data for the Lancashire and Greater Manchester footprint. This geography includes the majority of the catchment area for the PED in our study. Due to the age-range in our sample (2 months – 5 years) it was not possible to mirror the 5-year-old denominator methodology used for national samples (total number of children reaching their 5th birthday within the evaluation dates), we therefore used children aged 4yrs 0 months – 4 years 12 months as a proxy for 5-year-old data.

We compared the coverage figures for children in our study with local and national data obtained from the COVER programme. Chi-squared tests were used to examine differences in proportions and generate significance levels.

Results

A total of 1,450 children under the age of 5 years attended the PED in October 2021. Of these, 113 were under 2 months old (approximately 8 weeks, the age at which first tetanus-containing vaccines are given) so were excluded. For the remaining 1,337 children, records were available for 1,223 (91%) of them and are included in subsequent analyses. Children in the under-5s age group made up around 60% of all attendees to the PED (aged < 16 years at the time of presentation) in October 2021.

Participants

As the main variable of interest was attendance at the PED, no data beyond age were collected. The age distribution of children across ages was roughly uniform.

Tetanus-containing and MMR combined as a proxy for overall vaccination status

For peer review only

At the time of their presentation to the PED, two thirds of the 1,223 children had received all of their age-appropriate tetanus-containing and MMR vaccines (n = 807; 66.0%). This effectively equates to 416 missed opportunities to identify under-vaccinated children during their attendance within this sample.

Vaccination status varied by age band, with younger age groups tending to have higher levels of coverage (Figure 1, supplementary file 1). There were notable “dips” within the age bands where vaccines were due, for example MMR1 falls within the band 12m to <15m and coverage in this band was 70.3%, compared to 96.1% in the preceding band and 80.3% in the subsequent band. As a group, four-year-olds had very low levels of tetanus and MMR coverage, with just 1 in 5 of them having received a full set of primary and booster doses of tetanus plus two MMRs. Twenty-nine children in the sample appeared to be wholly unvaccinated.

Tetanus-containing vaccination

In the sample of children included in this study, 85.9% had received all age-appropriate tetanus-containing vaccinations at the time of presentation at the PED. Again, this varied by age group with higher levels of coverage present in children eligible only for their primary tetanus vaccines and coverage dropping in children from the age of 3 years 4 months when the “pre-school” booster vaccine is due.MMR vaccination

MMR1

During October 2021, 976 children attending PED were eligible for their first dose of the MMR vaccination. Of these, 640 (65.6%) had received the immunisation, meaning one third of children had not received their MMR vaccine despite being eligible (Figure 1).

For peer review only

MMR2

Of the 339 eligible children (older than 3 years and 4 months), only 68 had received their second MMR vaccination (20.1%). This was lowest in the youngest age band (3years 4 months – 3 years 6 months), with only 5.6% already having received the vaccination before their PED attendance. However, this rate remained low, even in the oldest children, e.g. only 14% of the children aged 4years – 4years and 3 months had received their MMR2 vaccine (Figure 1).

Estimate of "missing" tetanus and MMR vaccinations in a year

To estimate the total number of "missing" vaccinations amongst children under the age of five attending the study PED in a year, we assumed that the attendance figures for October 2021 were representative of a typical month in 2021 (recognising that pandemic-related restrictions and impacts were ongoing throughout 2021 and PED attendance would usually fluctuate across the year). Scaling up, it is estimated that a total of 4,992 children would have attended the study PED in 2021 with at least one missing tetanus or MMR vaccination. Specifically, if PED teams were equipped to administer catch up vaccinations (and all children were eligible and all parents/carers consented), up to 4,032 MMR1s, 3,252 MMR2s, and 1,404 tetanus boosters could potentially have been administered during 2021. Vaccines could also have been given to start, continue, or complete 960 primary tetanus courses (which would have required varying numbers of additional doses depending on the age of the child).

Comparison with National COVER data

Comparisons between publicly available data for the catchment area (Greater Manchester and Lancashire) and the children in our sample found no statistically

For peer review only

significant differences in coverage at 12 and 24 months (Table 1) or for the receipt of the full primary course of tetanus-containing vaccines by the age 5 years.

However, uptake of MMR1, MMR2, and the tetanus-containing booster (all by the age of 5 years) were all significantly lower in our sample of children than the general local population ($p<0.0001$).

	12 months		24 months		5 years	
	**L&GM**	**PED**	**L&GM**	**PED**	**L&GM**	**PED** [3]
Tetanus-containing primary course vaccine [1]	91.5	92.4	93.7	96.0	95.1	96.1
MMR1			90.3	90.9	94.6	34.2*
MMR2					87.0	21.7*
Tetanus-containing booster [2]					84.6	64.7*

[1] COVER data reports DTaP/IPV/Hib/HepB3

[2] COVER data reports Diphtheria, Tetanus, Polio, Pertussis Hib booster

[3] Children aged 4yr0mo - 4yr12mo used as a proxy for 5-year-olds

*Statistically significant difference in proportions of children vaccinated between L&GM and PED $p<0.0001$

Table 1. Proportion of children vaccinated and comparison of sample data (PED) with 2021-2022 COVER data for Lancashire and Greater Manchester (L&GM).

Finally, we compared the coverage in our sample to data for England at age five years. The children in our study had a higher proportion of coverage for the primary tetanus containing vaccine than the English sample (0.9% higher), however this was not statistically significant. A significantly lower proportion of children in our study were vaccinated with MMR1, MMR2, and the tetanus-containing booster vaccine than in the 2021-22 English cohort (60.4%, 65.3%, and 19.9% less respectively; all $p<0.0001$).

For peer review only

Discussion

The World Health Organization (WHO) recommends that at least 95% of children should be vaccinated against diseases preventable by immunisation to ensure elimination and control (24). Our data suggests that the children attending this PED in Greater Manchester were below this recommendation for MMR1 and 2, and for the tetanus-containing booster.

Principal findings

In the month of October 2021, 416 children (34% of those attending) were not up-to-date with their routine vaccinations at the time of their PED attendance. This equates to almost 5,000 potential vaccination moments in a single large PED – a currently unutilised opportunity to address unmet vaccination need, particularly amongst those experiencing vaccination inequity. MMR vaccination uptake in this PED-attending population was particularly low in older age groups. Whilst some of this is likely due to pandemic-related disruption, the finding that tetanus booster uptake, whilst low, was not as low as MMR2 uptake (despite them being offered during the same appointment), suggests that there may be something else contributing to the very low MMR2 numbers.

Strengths and weaknesses of the study

Whilst not in the original study design, a particular strength of this work is that it captured data within the pandemic and has provided additional insights into sub-populations of children who are now very under-immunised. Due to the pandemic, the study was restricted to a single site as the second study site (a local children's hospital) was not able to open to recruitment.

For peer review only

As with all studies of this type, there were issues around data quality and access, with around 10% of potential participants excluded due to inaccessible data. There were some obvious errors and inaccuracies within the SCR data (e.g. too many vaccines recorded or “immunisations given” with no further detail), which may have contributed to over- or under-estimation of vaccination status.

A proxy was used for overall vaccination status (tetanus and MMR combined) which is not ideal, but will only have led to an underestimate of the unmet vaccination need of children presenting to the PED. During the course of the work it became apparent that trying to find meaningful comparators for the study data would be tricky as national data are presented by individual vaccine type with no data available as to children’s overall vaccination status i.e. whether or not they are up-to-date with all their age-appropriate vaccinations. For the comparison at five years of age, we used a slightly younger cohort than the COVER data. However, as vaccination coverage rates locally, regionally, and nationally usually only increase by around 5% between the ages of 2 and 5 years (4), this is not likely to have led to a change in the statistical significance of the results, as the coverage at age 5 for everything except primary tetanus was so low.

Meaning of the study and implications

Under-5s attending the PED have unmet vaccination need, particularly amongst those eligible for MMR2 and tetanus boosters, and uptake of these so-called “pre-school boosters” differed by vaccine type.

Although there was no difference in vaccination coverage between the younger PED attendees and their peers in the local general population, they still had uptake levels below the “ideal” of 100% and mostly below the 95% target suggested by the WHO.

For peer review only

If one or more interventions were available to deliver to these under-vaccinated children during a PED attendance, hundreds of thousands of additional potential vaccine opportunities would be available to practitioners every year. Any future local or national campaigns e.g. “catch-up” for MMR, could look to utilise the PED as a potential site for delivery of vaccinations.

Future research

Future research will look at those aged 5 and older, to see if the patterns of unmet vaccination need are sustained in these other age groups, or if they are a pandemic-related phenomenon (or a combination of both). The cohort identified in this study as being under-immunised for tetanus and MMR might benefit from a “catch-up” campaign, and the PED would seem to be a potential additional location for this to be delivered. Future work will also look to co-design one or more interventions to support MMR catch-up in this age group, as well as exploring the potential for the development of other vaccination-focused programmes that could be delivered in the case of an outbreak e.g. mumps, or for routine vaccination e.g. influenza.

Having identified this under-immunised population attending the PED, it may be that there are other under-immunised children and young people attending other settings within the hospital who might benefit from the offer of a vaccination-focussed intervention. Identification of these populations e.g. those attending outpatient clinics, would facilitate more opportunities to offer vaccination interventions as part of routine hospital care.

Funding statement

This work received no direct funding, but RI was supported by a Health Education England Topol Digital Health Fellowship from February 2021 for a year.

For peer review only

Competing interests statement

None declared.

Author contributions

RI conceived of the study, led on the ethics application process, undertook the fieldwork, analysed the data, was involved in all stages of the preparation of this manuscript and is guarantor author. LB analysed the data and was involved in all stages of the preparation of this manuscript. FE supported data extraction and was involved in later drafts of the manuscript. ND and JK were involved in oversight of the project and were involved in later drafts of the manuscript.

Data sharing agreement

Relevant data may be requested from the lead author and will be provided in anonymised format as soon as legally and ethically possible.

Ethics approval statement

Full prospective ethics approval was obtained from the North West – Greater Manchester East Ethics Committee (IRAS reference 278815, REC reference 20/NW/0423). Following an earlier part of the work, an ethics amendment was submitted and approved to move to "opt-out" consent for this part of the project (Amendment 1, substantial, non-CTIMP, approved 26 August 2021).

For peer review only

References

1. Plotkin S. History of vaccination. *Proc Natl Acad Sci*. 2014 Aug 26;111(34):12283–7.

2. Smallpox [Internet]. [cited 2022 Jul 1]. Available from: https://www.who.int/westernpacific/health-topics/smallpox

3. Watson OJ, Barnsley G, Toor J, Hogan AB, Winskill P, Ghani AC. Global impact of the first year of COVID-19 vaccination: a mathematical modelling study. *Lancet Infect Dis*. 2022 Jun 23 [cited 2022 Jul 1]. Available from: https://www.thelancet.com/journals/laninf/article/PIIS1473-3099(22)00320-6/fulltext

4. Childhood Vaccination Coverage Statistics - 2021-22. NHS Digital. [cited 2023 Jan 13]. Available from: Childhood Vaccination Coverage Statistics- England, 2021-22 - NDRS (digital.nhs.uk)

5. Vaccination coverage for the second dose of measles-containing vaccine, EU/EEA, 2018 [Internet]. European Centre for Disease Prevention and Control. 2021 [cited 2022 Jul 1]. Available from: https://www.ecdc.europa.eu/en/publications-data/vaccination-coverage-second-dose-measles-containing-vaccine-eueea-2018

6. Hospital Episode Statistics » A&E Attendances and Emergency Admissions. [cited 2022 Jul 1]. Available from: https://www.england.nhs.uk/statistics/statistical-work-areas/ae-waiting-times-and-activity/

7. Isba R, Edge R, Jenner R, Broughton E, Francis N, Butler J. Where have all the children gone? Decreases in paediatric emergency department attendances at the start of the COVID-19 pandemic of 2020. *Arch Dis Child.* 2020 Jul 1;105(7):704–704.

8. Isba R, Edge R, Auerbach M, Cicero MX, Jenner R, Setzer E, et al. COVID-19. *Pediatr Emerg Care.* 2020. 36; 11: 551-553 [cited 2022 Jul 1]; Available from: https://www.ncbi.nlm.nih.gov/pmc/articles/PMC7493767/

9. Jenner R, Walker A, Isba R. Kids are back in town: the return of high demand for paediatric emergency care. *Arch Dis Child.* 2022 Feb 1;107(2):204–5.

10. Isba R, Edge R. Delivery of a multi-focus public health intervention in the paediatric emergency department: a feasibility and acceptability pilot study. *BMJ Open*. 2021 Dec 1;11(12):e047139.

11. Public Health England. National Immunisation Programme: health equity audit. February 2021.

12. Bladgen S, Newell K, Ghazarians N, Odunala M, Sulaiman S, Tunn L, et al. Interventions delivered in secondary or tertiary medical care settings to improve routine vaccination uptake in children and young people: a scoping review. *BMJ Open*. Accepted with minor revisions.

For peer review only

13. Cunningham SJ. Providing immunizations in a pediatric emergency department: underimmunization rates and parental acceptance. *Pediatr Emerg Care.* 1999;15(4):255–9.

14. Bell LM, Pritchard M, Anderko R, Levenson R. A Program to Immunize Hospitalized Preschool-aged Children: Evaluation and Impact. *Pediatrics*. 1997 Aug 1;100(2):192–6.

15. Tarca AJ, Lau GT, Mascaro F, Clifford P, Campbell AJ, Taylor E. Pre- and post-intervention study examining immunisation rates, documentation, catch-up delivery and the impact of a dedicated immunisation service at a tertiary paediatric hospital. *J Paediatr Child Health*. 2021;57(2):263–7.

16. Hutchison RL, O'Rear J, Olson-Burgess C, Myers AL. Offering the Influenza Vaccine in a Pediatric Hand Surgery Clinic Increases Vaccination Rates. *J Hand Surg*. 2018 Aug 1;43(8):776.e1-776.e4.

17. Cameron MA, Bigos D, Festa C, Topol H, Rhee KE. Missed Opportunity: Why Parents Refuse Influenza Vaccination for Their Hospitalized Children. *Hosp Pediatr.* 2016 Sep 1;6(9):507–12.

18. Child and Maternal Health - PHE. [cited 2021 Feb 6]. Available from: https://fingertips.phe.org.uk/profile/child-health-profiles/data#page/0/gid/1938133228/pat/6/par/E12000002/ati/202/are/E08000003/iid/93700/age/169/sex/4/cid/4/tbm/1/page-options/ovw-do-0

19. GOV.UK Complete routine immunisation schedule. [cited 2022 Jul 1]. Available from: https://www.gov.uk/government/publications/the-complete-routine-immunisation-schedule

20. Isba R. Should we use Summary Care Records to access vaccination data in the Paediatric Emergency Department? Child Health Technology Conference. 2022.

21. Childhood Vaccination Coverage Statistics - England 2018-19. NHS Digital. [cited 2022 Jul 1]. Available from: https://digital.nhs.uk/data-and-information/publications/statistical/nhs-immunisation-statistics/england-2018-19

22. Stata: Software for Statistics and Data Science. [cited 2022 Jul 1]. Available from: https://www.stata-uk.com/software/stata.html/?utm_medium=adwords&utm_campaign=&utm_source=&gclid=Cj0KCQjwtvqVBhCVARIsAFUxcRvI4U2isNRQO_OqwQ6soXemgNuKF82amyEUsbw5v7cmOq2Hwhtvx4caApvAEALw_wcB

23. NHS Digital. Appendices - coverage definitions. [cited 2022 Jul 1]. Available from: https://digital.nhs.uk/data-and-information/publications/statistical/nhs-immunisation-statistics/england---2020-21/appendices#appendix-b-coverage-definitions

24. World Health Organization. Health21: the health for all policy framework for the WHO European Region. Copenhagen: World Health Organization, Regional Office for Europe; 1999. 224 p. (European health for all series).

For peer review only

For peer review only

Figure 1 - Level of vaccination coverage by age

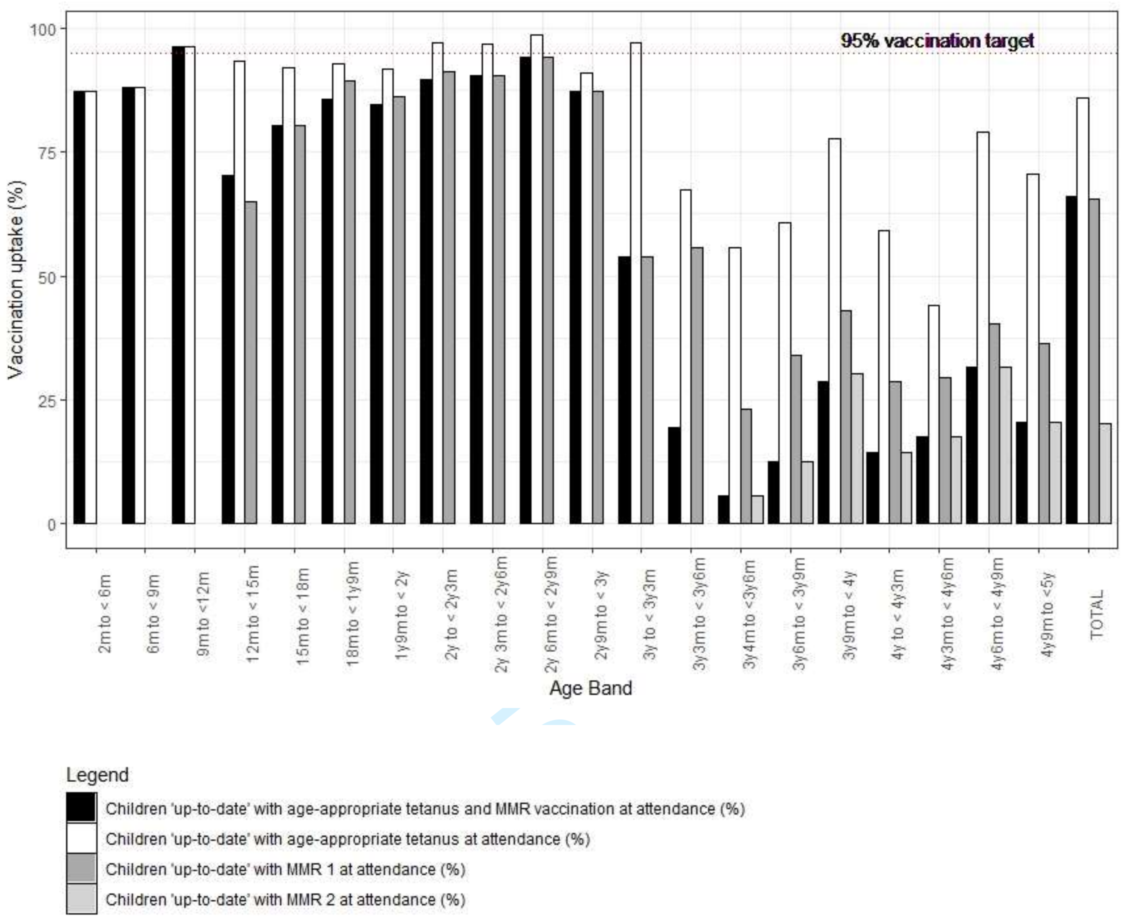

*Data to support figure 1 is included in supplementary file 1

Supplementary file 1 – Data to support figure 1

Age band	Total number of children attending	Total number of children attending with vaccination data available	Children 'up-to-date' with age-appropriate tetanus and/or MMR vaccination at attendance (n (%))	Children 'up-to-date' with age-appropriate tetanus at attendance (n (%))	Children 'up-to-date with MMR 1 at attendance (n (%))	Up to date with MMR 2 at attendance
2m to < 6m	91	79	69 (87.3)	69 (87.3)		
6m to < 9m	74	66	58 (87.9)	58 (87.9)		
9m to <12m	113	102	98 (96.1)	98 (96.1)		
12m to < 15m	80	74	52 (70.3)	69 (93.2)	48 (64.9)	
15m to < 18m	86	76	61 (80.3)	70 (92.1)	61 (80.3)	
18m to < 1y9m	90	84	72 (85.7)	78 (92.9)	75 (89.3)	
1y9m to < 2y	77	72	61 (84.7)	66 (91.7)	62 (86.1)	
2y to < 2y3m	72	68	61 (89.7)	66 (97.1)	62 (91.2)	
2y 3m to < 2y6m	68	62	56 (90.3)	60 (96.8)	56 (90.3)	
2y 6m to < 2y9m	71	68	64 (94.1)	67 (98.5)	64 (94.1)	
2y9m to < 3y	62	55	48 (87.3)	50 (90.9)	48 (87.3)	
3y to < 3y3m	70	65	35 (53.8)	63 (96.9)	35 (53.8)	
3y3m to < 3y6m	53	49	7 (19.4)	33 (67.3)	20 (55.6)	
3y4m to <3y6m	*39*	*36*	*2 (5.6)**	*20 (55.6)*	*9 (23.1)*	*2 (5.6)*
3y6m to < 3y9m	60	56	7 (12.5)	34 (60.7)	19 (33.9)	7 (12.5)
3y9m to < 4y	69	63	18 (28.6)	49 (77.8)	27 (42.9)	19 (30.2)
4y to < 4y3m	53	49	7 (14.3)	29 (59.2)	14 (28.6)	7 (14.3)
4y3m to < 4y6m	39	34	6 (17.6)	15 (44.1)	10 (29.4)	6 (17.6)
4y6m to < 4y9m	59	57	18 (31.6)	45 (78.9)	23 (40.4)	18 (31.6)
4y9m to <5y	50	44	9 (20.5)	31 (70.5)	16 (36.4)	9 (20.5)
TOTAL	1337	1259	807 (66.0)	1050 (85.9)	640 (65.6)	68 (20.1)

*Denominator for calculation is number of children aged 3y4m < 3yr 6m (n=36)

For peer review only

Supplementary file 1 – Data to support figure 1

For peer review only

STROBE Statement—Checklist of items that should be included in reports of ***cross-sectional studies***

	Item No	Recommendation	Page No
**Title and abstract**	1	(*a*) Indicate the study's design with a commonly used term in the title or the abstract	1
		(*b*) Provide in the abstract an informative and balanced summary of what was done and what was found	2
**Introduction**			
Background/rationale	2	Explain the scientific background and rationale for the investigation being reported	4-6
Objectives	3	State specific objectives, including any prespecified hypotheses	6
**Methods**			
Study design	4	Present key elements of study design early in the paper	7
Setting	5	Describe the setting, locations, and relevant dates, including periods of recruitment, exposure, follow-up, and data collection	7
Participants	6	(*a*) Give the eligibility criteria, and the sources and methods of selection of participants	7
Variables	7	Clearly define all outcomes, exposures, predictors, potential confounders, and effect modifiers. Give diagnostic criteria, if applicable	9
Data sources/ measurement	8*	For each variable of interest, give sources of data and details of methods of assessment (measurement). Describe comparability of assessment methods if there is more than one group	9-10
Bias	9	Describe any efforts to address potential sources of bias	
Study size	10	Explain how the study size was arrived at	9
Quantitative variables	11	Explain how quantitative variables were handled in the analyses. If applicable, describe which groupings were chosen and why	10
Statistical methods	12	(*a*) Describe all statistical methods, including those used to control for confounding	10-11
		(*b*) Describe any methods used to examine subgroups and interactions	
		(*c*) Explain how missing data were addressed	
		(*d*) If applicable, describe analytical methods taking account of sampling strategy	
		(*e*) Describe any sensitivity analyses	
**Results**			
Participants	13*	(a) Report numbers of individuals at each stage of study—eg numbers potentially eligible, examined for eligibility, confirmed eligible, included in the study, completing follow-up, and analysed	11
		(b) Give reasons for non-participation at each stage	
		(c) Consider use of a flow diagram	
Descriptive data	14*	(a) Give characteristics of study participants (eg demographic, clinical, social) and information on exposures and potential confounders	11
		(b) Indicate number of participants with missing data for each variable of interest	11
Outcome data	15*	Report numbers of outcome events or summary measures	11
Main results	16	(*a*) Give unadjusted estimates and, if applicable, confounder-adjusted estimates and their precision (eg, 95% confidence interval). Make clear which confounders were adjusted for and why they were included	12-14

For peer review only

		(*b*) Report category boundaries when continuous variables were categorized	
		(*c*) If relevant, consider translating estimates of relative risk into absolute risk for a meaningful time period	
Other analyses	17	Report other analyses done—eg analyses of subgroups and interactions, and sensitivity analyses	14
**Discussion**			
Key results	18	Summarise key results with reference to study objectives	15
Limitations	19	Discuss limitations of the study, taking into account sources of potential bias or imprecision. Discuss both direction and magnitude of any potential bias	15-16
Interpretation	20	Give a cautious overall interpretation of results considering objectives, limitations, multiplicity of analyses, results from similar studies, and other relevant evidence	16-17
Generalisability	21	Discuss the generalisability (external validity) of the study results	17
**Other information**			
Funding	22	Give the source of funding and the role of the funders for the present study and, if applicable, for the original study on which the present article is based	17

*Give information separately for exposed and unexposed groups.

Note: An Explanation and Elaboration article discusses each checklist item and gives methodological background and published examples of transparent reporting. The STROBE checklist is best used in conjunction with this article (freely available on the Web sites of PLoS Medicine at http://www.plosmedicine.org/, Annals of Internal Medicine at http://www.annals.org/, and Epidemiology at http://www.epidem.com/). Information on the STROBE Initiative is available at www.strobe-statement.org.

For peer review only

BMJ Open

Unmet vaccination need amongst children under the age of five attending the Paediatric Emergency Department: a cross-sectional study in a large UK district general hospital.

Journal:	*BMJ Open*
Manuscript ID	bmjopen-2023-072053.R1
Article Type:	Original research
Date Submitted by the Author:	05-May-2023
Complete List of Authors:	Isba, Rachel; Lancaster University; Manchester University NHS Foundation Trust<br>Brennan, Louise; Lancaster University; Manchester University NHS Foundation Trust<br>Egboko, Fiona; Lancaster University<br>Edge, Rhiannon; Lancaster University<br>Davies, Nigel; Lancaster University<br>Knight, Joanne; Lancaster University
**Primary Subject Heading**:	Public health
Secondary Subject Heading:	Paediatrics
Keywords:	PUBLIC HEALTH, Paediatric infectious disease & immunisation < PAEDIATRICS, Public health < INFECTIOUS DISEASES

SCHOLARONE™
Manuscripts

I, the Submitting Author has the right to grant and does grant on behalf of all authors of the Work (as defined in the below author licence), an exclusive licence and/or a non-exclusive licence for contributions from authors who are: i) UK Crown employees; ii) where BMJ has agreed a CC-BY licence shall apply, and/or iii) in accordance with the terms applicable for US Federal Government officers or employees acting as part of their official duties; on a worldwide, perpetual, irrevocable, royalty-free basis to BMJ Publishing Group Ltd ("BMJ") its licensees and where the relevant Journal is co-owned by BMJ to the co-owners of the Journal, to publish the Work in this journal and any other BMJ products and to exploit all rights, as set out in our licence.

The Submitting Author accepts and understands that any supply made under these terms is made by BMJ to the Submitting Author unless you are acting as an employee on behalf of your employer or a postgraduate student of an affiliated institution which is paying any applicable article publishing charge ("APC") for Open Access articles. Where the Submitting Author wishes to make the Work available on an Open Access basis (and intends to pay the relevant APC), the terms of reuse of such Open Access shall be governed by a Creative Commons licence – details of these licences and which Creative Commons licence will apply to this Work are set out in our licence referred to above.

Other than as permitted in any relevant BMJ Author's Self Archiving Policies, I confirm this Work has not been accepted for publication elsewhere, is not being considered for publication elsewhere and does not duplicate material already published. I confirm all authors consent to publication of this Work and authorise the granting of this licence.

For peer review only

Unmet vaccination need amongst children under the age of five attending the Paediatric Emergency Department: a cross-sectional study in a large UK district general hospital.

Isba, Rachel [1,2] rachel.isba@lancaster.ac.uk (0000-0002-2896-4309), Professor
Brennan, Louise [1,2,3] louise.brennan8@nhs.net (0000-0003-0377-6720), Public Health Registrar
Egboko, Fiona [1] f.egboko@lancaster.ac.uk, Medical Student
Edge, Rhiannon [1] r.edge@lancaster.ac.uk (0000-0001-5349-783X), Lecturer
Davies, Nigel [4] n.a.davies@lancaster.ac.uk (0000-0002-4136-5300), Professor
Knight, Jo [1] jo.knight@lancaster.ac.uk (0000-0002-7148-1660), Professor

[1] Lancaster Medical School, Lancaster University, Lancaster, UK

[2] Manchester University NHS Foundation Trust, Manchester, UK

[3] St Helens and Knowsley Teaching Hospitals NHS Trust, St Helens, UK

[4] School of Computing and Communication, Lancaster University, Lancaster, UK

Corresponding Author: Rachel Isba rachel.isba@lancaster.ac.uk

For peer review only - http://bmjopen.bmj.com/site/about/guidelines.xhtml

Abstract

Objective

The objective was to estimate vaccination coverage amongst children under the age of five attending the Paediatric Emergency Department (PED) using tetanus and MMR vaccination as a proxy.

Design

A cross-sectional observational study with a single data collection point for each participant.

Setting

A single large PED in Greater Manchester, England.

Participants

Participants were children (under 5 years old) attending the PED during October 2021. Participation was "opt-out" and parents/carers were given until the end of the following month to request that their child's data be excluded.

Primary and secondary outcome measures

The primary outcome of interest was the percentage of children who were up-to-date with their routine childhood vaccinations at their time of attendance to the PED. Secondary outcome measures were the percentage of children who had received age-appropriate tetanus and MMR vaccination, and how these compared to local population data at the ages of 1, 2, and 5 years of age.

Results

One third of under-5s in this study had unmet vaccination need and were missing at least one dose of either MMR or tetanus-containing vaccine. In older age groups,

For peer review only - http://bmjopen.bmj.com/site/about/guidelines.xhtml

many were missing their tetanus boosters and only 1 in 5 of those eligible had received two doses of MMR. Those in younger age groups had vaccination coverage levels comparable to the local data, but still below the target of 95%.

Conclusions

Those children eligible for pre-school boosters (tetanus and MMR2) appear to have considerable unmet vaccination need. Whilst the pandemic has had an impact, the observation that MMR2 uptake is considerably lower than tetanus booster (when they are scheduled together) warrants further investigation. Catch-up campaigns for MMR2 should focus on this cohort of children and the PED may offer an opportunity for an intervention.

Trial registration

The study was registered as an observational/non-interventional study on the clinicaltrials.gov website with the identifier NCT04485624.

Word Count: 4079

Key words:
Public health, Paediatric infectious disease & immunisation, public health infectious diseases

For peer review only

Article Summary

Strengths and limitations of this study

- This study is timely given the pandemic's impact on routine childhood vaccination.
- A population has been identified with considerable unmet vaccination need who might benefit from an intervention not currently offered.
- A limitation is that the work was undertaken at a single centre and there were constraints with data collection and quality.
- A proxy for overall vaccination status was used, leading to a possible underestimation of unmet vaccination need.

For peer review only

For peer review only - http://bmjopen.bmj.com/site/about/guidelines.xhtml

Introduction

Vaccines are one of the great global health successes. Since their discovery more than 300 years ago, vaccines have saved countless millions of lives (1), reduced the incidence of dozens of diseases, and even lead to the eradication of smallpox (2). In countries with access to them, vaccines have also played a key part in the control of the current SARS-CoV-2/COVID-19 pandemic (3).

However, pre-pandemic, in the UK, uptake of routine childhood vaccination had fluctuated (4) and coverage lagged behind some of our European peers for common vaccine-preventable diseases such as measles (5).

Every year in England, millions of children and young people (CYP) attend hospital (secondary or tertiary medical care) (6). Attendance is often with relatively minor illnesses and injuries, many of which could be better managed elsewhere. However, despite numerous initiatives to redirect these CYP, hospital attendances (pre-pandemic) had increased year on year (6). The pronounced decrease in Paediatric Emergency Department (PED) attendances seen early in the pandemic (7,8) was reversed in 2021, as lockdowns and other restrictions were eased, with attendance exceeding pre-pandemic levels and a change in the some of the seasonal patterns of illnesses presenting to the PED (9).

In addition to their primary reason for presentation, CYP attending the hospital may have lower than average levels of health and wellbeing, additional unmet health need (e.g. dental health), or not be able to engage with preventive elements of routine healthcare (e.g. vaccination) for a myriad of reasons. A hospital attendance or admission might therefore offer an opportunity to intervene. A recent pilot study showed that time during a PED department attendance could be used to deliver a

For peer review only

For peer review only - http://bmjopen.bmj.com/site/about/guidelines.xhtml

public health intervention and that this was both feasible (within the constraints of the department) and acceptable (to all stakeholders including CYP, parents and carers, and staff in the PED) (10).

If any child or young person who had not received their age-appropriate routine vaccinations could be identified during a PED attendance, clinicians might (should it be clinically/situationally appropriate) be able to offer one or more tailored interventions to address this unmet vaccination need. The benefits of such an approach are numerous and include:

- decreasing mortality and morbidity from vaccine-preventable diseases, by ensuring
    - individual and population coverage for diseases that cannot spread person-person e.g. tetanus
    - higher levels of population coverage for non-epidemic diseases that can be spread person-person e.g. Hepatitis B
    - and herd immunity for diseases that can easily spread person-person and can cause outbreaks e.g. measles;
- a decrease in un-needed treatment in the case of individual exposure in the absence of an accurate vaccination history at the point of treatment e.g. a tetanus-prone wound in the PED;
- a reactive response to outbreaks e.g. mumps, epidemics e.g. influenza, and pandemics e.g. SARS-CoV-2;
- improving coverage of targeted vaccination programmes e.g. seasonal influenza.

For peer review only - http://bmjopen.bmj.com/site/about/guidelines.xhtml

In the UK the routine immunisation schedule is commissioned nationally. Vaccinations are free and parents/carers of children are invited and reminded about vaccinations. Pre-school this is done through General Practice, whilst school-age services are delivered through School Immunisation Teams. Immunisations are recorded in Primary Care Records (maintained by General Practitioners) and the Child Health Information Service (CHIS). In some regions these vaccination records are not accessible in secondary care, however some areas have access to Summary Care Records (SCRs) which is a centralised NHS computer system containing information such as patient medications and allergies (11). Some regions of the UK have enhanced SCRs containing vaccination data. Regardless of whether SCRs are available, the vaccination status of a child should be checked with the caregiver as part of routine care within these settings (12). Whilst vaccines are not mandatory, they are recommended, and the Department of Health and Social Care take responsibility for the vaccination strategy and initiation of promotion/ uptake campaigns (13). In its 2021 Health Equity Audit of the National Immunisation Programme (14), Public Health England (now the UK Health Security Agency) stated “Equality in immunisation is an important way to address health inequalities” and reported that whilst the Immunisation Programme had achieved high coverage in the population as a whole, within sub-populations there still existed “avoidable inequalities”. Whilst the reasons for these inequalities are complex, “institutional” and “policy” factors play a role (14). Addressing these factors by offering vaccination at the point of PED attendance may preferentially improve vaccination uptake amongst those experiencing avoidable inequalities.

Previous work to improve vaccination uptake via interventions delivered in secondary or tertiary care (see (15) for overview) has shown that vaccination coverage in CYP

For peer review only - http://bmjopen.bmj.com/site/about/guidelines.xhtml

attending hospital settings is generally lower than in the general population. However, the vaccinations under investigation, location of presentation within the hospital, and way in which vaccination data were verified, varies considerably. In the previous study (15), for all due/overdue vaccinations (not a measure currently available in England), baseline coverage ranged from 44% (16,17) – 89% (18), with little difference by setting and a trend for lower coverage in older studies. For influenza, baseline coverage was lower, ranging from 25% (19) - 50.5% (20).

The overall aim of this work was to answer the question: Do children (aged < 5 years) attending the PED have lower levels of vaccination coverage than their peers in the general population? This allowed us to assess the unmet vaccination need of children attending the PED.

Methods

This was a cross-sectional observational study with a single data collection point for each child. The study was registered as an observational/non-interventional study on the clinicaltrials.gov website with the identifier NCT04485624.

Consent

Full prospective ethics approval was obtained from the North West – Greater Manchester East Ethics Committee (IRAS reference 278815, REC reference 20/NW/0423). Following an earlier part of the work, an ethics amendment was submitted and approved to move to “opt-out” consent for this part of the project (Amendment 1, substantial, non-CTIMP, approved 26 August 2021).

Prominently-displayed posters relating to the study were put up around the PED during the month of data collection, with flyers handed out in triage, and participant information sheets with opt-out consent forms available on request. Nursing staff in

For peer review only - http://bmjopen.bmj.com/site/about/guidelines.xhtml

the PED were briefed so that they could support queries from parents/carers. Parents/carers could choose for their child to opt-out by filling in the form or contacting a member of the research team within a month of attendance.

Setting

Data were collected from a single, dedicated co-located PED, in Greater Manchester, in the North West of England.

Participants

Participants were children (under 5 years old) attending the PED during October 2021. Children and young people in Manchester have lower than average levels of health and wellbeing, around a third live in low-income households, and 1 in 100 of them reside in care (21).

Patient and public involvement

Reading materials were designed to be read by the parent or caregiver. Following patient involvement in a previous study (10) all reading materials were designed for a reading age of 7-9 years old, in order to be as accessible as possible.

Vaccination schedule

The routine NHS vaccination schedule (22) recommends tetanus-containing vaccines (given as multi-component vaccines) are given at 8, 12, and 16 weeks ("primary course") and at around 3 years 4 months (as part of the so-called "pre-school booster"). MMR1 is due around one year of age and MMR2 is given at 3 years 4 months, alongside the pre-school booster.

For peer review only - http://bmjopen.bmj.com/site/about/guidelines.xhtml

Data collection

A list of dates of birth and hospital numbers was generated for all attendees during the month of October 2021. Duplicates (where the same child may have attended more than once in the month of interest) were removed. Exact age at presentation was not available, so was calculated based on age in completed months on October 1st 2021. Any vaccination before the end of October 2021 was included when checking status. This approach was taken to ensure that the data tended to over- rather than under-estimate coverage e.g. at the lower age limits.

Data were extracted from individual Summary Care Records (SCRs) by RI and anonymised before analysis. In Greater Manchester, SCRs contain information on the child's health and vaccination status. Extraction took between 2 and 5 minutes per record (depending on the quality of network access/connectivity). The quality and presentation of the data within the SCR was also very variable and this finding is presented elsewhere (23).

Sample size calculation

The MMR vaccine was chosen for the sample size calculation as this is the vaccine that has attracted the most controversy over the past decades (resulting in lower than target coverage).

At the time the sample size calculation was carried out, the most recent data for MMR2 coverage at 5 years were available for the year 2018/19 (24). In England, coverage for MMR2 was 86.4% and for Manchester, coverage for 5 year olds was 82.1%. The sample size calculation was carried out using STATA version 16 (25) and a comparison made between population prevalence (between the "PED" and

For peer review only - http://bmjopen.bmj.com/site/about/guidelines.xhtml

"general Manchester" populations). The sample size calculation was based on the difference between the population prevalence in Manchester of 0.82 and in the study PED of 0.77 (i.e. assumed to be 5% less than the general population), with the required power at 0.8, using a two-sided test, with alpha level set at $p < 0.05$. This resulted in an estimated sample size for a one-sample proportion test (Wald z test) of 577.

Outcomes

Primary

The primary outcome of interest was the percentage of children aged > 2 months (to reflect the start of the immunisation schedule in the UK) and < 5 years (the age at which UK COVER data assesses vaccination uptake) who were "up to date" with all their age-appropriate tetanus-containing and MMR vaccinations at the time of PED attendance (this was used as a proxy for overall vaccination status);

Children were coded as "up to date" with their vaccinations if they had received all tetanus and MMR vaccinations for which they were eligible (based on their age) by 31st October 2021.

Secondary

Secondary outcomes of interest were age-appropriate tetanus-containing vaccination coverage, and uptake of MMR1 and MMR2. Specifically:

- > 2 months and < 5 years who had received all their age-appropriate tetanus vaccinations at the time of PED attendance;
- > 12 months and < 5 years who had received at least one dose of MMR at the time of PED attendance;

For peer review only - http://bmjopen.bmj.com/site/about/guidelines.xhtml

- between 2 and < 5 years who had received MMR1 by their 2nd birthday;
- between 3 years 4 months and < 5 years who had received two doses of MMR at the time of PED attendance;
- between 3 years 4 months and < 5 years who had received MMR2 by their 5th birthday.

Statistical Analysis

Descriptive statistics were prepared for primary and secondary outcomes. We compared our data to data published for the year April 2021 - 2022 in the cover of vaccination evaluated rapidly (COVER) programme (4). The COVER programme publishes quarterly and annual vaccination coverage statistics for children aged 12 months, 24 months, and 5 years in the UK. Data from 2021-22 was chosen as they were the most recent published data and to correspond with the study period.

We mirrored the methodology used for the COVER data (26) using number of eligible persons immunised as the numerator and total number of persons in the eligible population as the denominator, to obtain a comparable sample from our study in the PED. We compared our data at 12 months, 24 months, and 5 years with the publicly available data for the Lancashire and Greater Manchester footprint. This geography includes the majority of the catchment area for the PED in our study. Due to the age-range in our sample (2 months – 5 years) it was not possible to mirror the 5-year-old denominator methodology used for national samples (total number of children reaching their 5th birthday within the evaluation dates), we therefore used children aged 4yrs 0 months – 4 years 12 months as a proxy for 5-year-old data.

For peer review only - http://bmjopen.bmj.com/site/about/guidelines.xhtml

We compared the coverage figures for children in our study with local and national data obtained from the COVER programme. Chi-squared tests were used to examine differences in proportions and generate significance levels.

Results

A total of 1,450 children under the age of 5 years attended the PED in October 2021. Of these, 113 were under 2 months old (the age at which first tetanus-containing vaccines are given) and were therefore excluded. For the remaining 1,337 children, records were available for 1,223 (91%) of them and are included in subsequent analyses. Records were unavailable if it was not possible to see the vaccination data, e.g. children living outside Greater Manchester had no GM summary care record. No parents/carers chose to 'opt-out' of the study. Children in the under-5s age group made up around 60% of all attendees to the PED (aged < 16 years at the time of presentation) in October 2021.

Participants

As the main variable of interest was attendance at the PED, no data beyond age were collected. The age distribution of children across ages was roughly uniform (supplementary file 1).

Tetanus-containing and MMR combined as a proxy for overall vaccination status

At the time of their presentation to the PED, two thirds of the 1,223 children had received all of their age-appropriate tetanus-containing and MMR vaccines (n = 807; 66.0%). This effectively equates to 416 missed opportunities to identify under-vaccinated children during their attendance within this sample.

For peer review only - http://bmjopen.bmj.com/site/about/guidelines.xhtml

Vaccination status varied by age band, with younger age groups tending to have higher levels of coverage (Figure 1, supplementary file 2). There were notable "dips" within the age bands where vaccines were due, for example MMR1 falls within the band 12m to <15m and coverage in this band was 70.3%, compared to 96.1% in the preceding band and 80.3% in the subsequent band. As a group, four-year-olds had very low levels of tetanus and MMR coverage, with just 1 in 5 of them having received a full set of primary and booster doses of tetanus plus two MMRs. Twenty-nine children in the sample had no vaccinations recorded, e.g. the parent/ caregiver had not given consent for vaccinations and this was recorded in the SCR.

Tetanus-containing vaccination

In the sample of children included in this study, 85.9% had received all age-appropriate tetanus-containing vaccinations at the time of presentation at the PED. Again, this varied by age group with higher levels of coverage present in children eligible only for their primary tetanus vaccines and coverage dropping in children from the age of 3 years 4 months when the "pre-school" booster vaccine is due.

MMR1

During October 2021, 976 children attending PED were eligible for their first dose of the MMR vaccination. Of these, 640 (65.6%) had received the immunisation, meaning one third of children had not received their MMR vaccine despite being eligible (Figure 1).

MMR2

Of the 339 eligible children (older than 3 years and 4 months), only 68 had received their second MMR vaccination (20.1%). This was lowest in the youngest age band (3years 4 months – 3 years 6 months), with only 5.6% already having received the

For peer review only - http://bmjopen.bmj.com/site/about/guidelines.xhtml

vaccination before their PED attendance. However, this rate remained low, even in the oldest children, e.g. only 14% of the children aged 4 years – 4 years and 3 months had received their MMR2 vaccine, and 20.5% in children aged 4 years and 9 months and 5 (Figure 1).

Comparison with National COVER data

Comparisons between publicly available data for the catchment area (Greater Manchester and Lancashire) and the children in our sample found no statistically significant differences in coverage at 12 and 24 months (Table 1) or for the receipt of the full primary course of tetanus-containing vaccines by the age 5 years.

However, uptake of MMR1, MMR2, and the tetanus-containing booster (all by the age of 5 years) were all significantly lower in our sample of children than the general local population (p<0.0001).

	12 months		24 months		5 years	
	**L&GM**	**PED**	**L&GM**	**PED**	**L&GM**	**PED [3]**
Tetanus-containing primary course vaccine [1]	91.5 (44,195)	92.4 (283)	93.7 (47,600)	96.0 (243)	95.1 (53,541)	96.1 (177)
MMR1			90.3 (43,615)	90.9 (230)	94.6 (53,260)	34.2* (63)
MMR2					87.0 (48,981)	21.7* (40)
Tetanus-containing booster [2]					84.6 (47,630)	64.7* (119)

[1] COVER data reports DTaP/IPV/Hib/HepB3

[2] COVER data reports Diphtheria, Tetanus, Polio, Pertussis Hib booster

[3] Children aged 4yr0mo - 4yr12mo used as a proxy for 5-year-olds

*Statistically significant difference in proportions of children vaccinated between L&GM and PED p<0.0001

Table 1. Proportion (and number) of children vaccinated and comparison of sample data (PED) with 2021-2022 COVER data for Lancashire and Greater Manchester (L&GM).

For peer review only - http://bmjopen.bmj.com/site/about/guidelines.xhtml

Finally, we compared the coverage in our sample to data for England at age five years. The children in our study had a higher proportion of coverage for the primary tetanus containing vaccine than the English sample (0.9% higher), however this was not statistically significant. A significantly lower proportion of children in our study were vaccinated with MMR1, MMR2, and the tetanus-containing booster vaccine than in the 2021-22 English cohort (60.4%, 65.3%, and 19.9% less respectively; all p<0.0001). Whilst there may be lag noted in some of our data (where children have a low rate of coverage in the age bands immediately after the age at which their vaccine is first due), this is unlikely to impact comparisons with the COVER data as the comparator points are not around the age at which vaccines are due.

Discussion

The World Health Organization (WHO) recommends that at least 95% of children should be vaccinated against diseases preventable by immunisation to ensure elimination and control (27). Our data suggests that the children attending this PED in Greater Manchester were below this recommendation for MMR1 and 2, and for the tetanus-containing booster.

When comparing our PED population to their peers in the general population we found that there were no statistically significant differences in coverage at age 12 and 24 months. However, uptake of MMR1 and MMR2 and the tetanus containing booster at age 5 were significantly lower in the PED than in the general population. This study supports previous findings (14) and suggests that the PED is an opportune location for vaccine interventions (10).

For peer review only - http://bmjopen.bmj.com/site/about/guidelines.xhtml

Estimate of "missing" tetanus and MMR vaccinations in a year

To estimate the total number of "missing" vaccinations amongst children under the age of five attending the study PED in a year, we assumed that the attendance figures for October 2021 were representative of a typical month in 2021 (recognising that pandemic-related restrictions and impacts were ongoing throughout 2021 and PED attendance would usually fluctuate across the year with higher rates in winter and lower rates occurring in summer). Scaling up, we estimated that a total of 4,992 children would have attended the study PED in 2021 with at least one missing tetanus or MMR vaccination. Specifically, if PED teams were equipped to administer catch up vaccinations (and all children were eligible and all parents/carers consented), up to 4,032 MMR1s, 3,252 MMR2s, and 1,404 tetanus boosters could potentially have been administered during 2021. Vaccines could also have been given to start, continue, or complete 960 primary tetanus courses (which would have required varying numbers of additional doses depending on the age of the child). It is recognised that these are approximations and could be overestimates e.g. as some children attending the PED will be too unwell to have an intervention.

Principal findings

In the month of October 2021, 416 children (34% of those attending) were not up-to-date with their routine vaccinations at the time of their PED attendance. This could equate to up to 5,000 potential vaccination moments in a single large PED – a currently unutilised opportunity to address unmet vaccination need, particularly amongst those experiencing vaccination inequity. MMR vaccination uptake in this PED-attending population was particularly low in older age groups, and significantly lower than the general population. Whilst some of this is likely due to pandemic-

For peer review only - http://bmjopen.bmj.com/site/about/guidelines.xhtml

related disruption (e.g. lockdowns), the finding that tetanus booster uptake, whilst low, was not as low as MMR2 uptake (despite them being offered during the same appointment), suggests that there may be something else contributing to the very low MMR2 numbers, and this would warrant further investigation in future studies.

Strengths and weaknesses of the study

Whilst not in the original study design, a particular strength of this work is that it captured data within the pandemic and has provided additional insights into sub-populations of children who are now very under-immunised. Due to the pandemic, the study was restricted to a single site as the second study site (a local children's hospital) was not able to open to recruitment.

As with all studies of this type, there were issues around data quality and access, with around 10% of potential participants excluded due to inaccessible data. There were some obvious errors and inaccuracies within the SCR data (e.g. too many vaccines recorded or "immunisations given" with no further detail), which may have contributed to over- or under-estimation of vaccination status.

This study took place in an unusual data ecosystem where access to a summary care record (and therefore vaccination status) was possible. This study may not be reproducible elsewhere and recommendations for interventions to investigate vaccination status within the PED would require access to this information. Access to reliable vaccination data in acute care settings remains an issue.

A proxy was used for overall vaccination status (tetanus and MMR combined) which is not ideal, but will only have led to an underestimate of the unmet vaccination need of children presenting to the PED. During the course of the work it became apparent that trying to find meaningful comparators for the study data would be tricky as

For peer review only

For peer review only - http://bmjopen.bmj.com/site/about/guidelines.xhtml

national data are presented by individual vaccine type with no data available as to children's overall vaccination status i.e. whether or not they are up-to-date with all their age-appropriate vaccinations. For the comparison at five years of age, we used a slightly younger cohort than the COVER data. However, as vaccination coverage rates locally, regionally, and nationally usually only increase by around 5% between the ages of 2 and 5 years (4), this is not likely to have led to a change in the statistical significance of the results, as the coverage at age 5 for everything except primary tetanus was so low.

Meaning of the study and implications

Under-5s attending the PED have unmet vaccination need, particularly amongst those eligible for MMR2 and tetanus boosters, and uptake of these so-called "pre-school boosters" differed by vaccine type.

Although there was no difference in vaccination coverage between the younger PED attendees and their peers in the local general population, they still had uptake levels below the "ideal" of 100% and mostly below the 95% target suggested by the WHO.

If one or more interventions were available to deliver to these under-vaccinated children during a PED attendance, hundreds of thousands of additional potential vaccine opportunities would be available to practitioners every year. Any future local or national campaigns e.g. "catch-up" for MMR, could look to utilise the PED as a potential site for delivery of vaccinations.

Future research

Future research will look at those aged 5 and older, to see if the patterns of unmet vaccination need are sustained in these other age groups, or if they are a pandemic-related phenomenon (or a combination of both). The cohort identified in this study as

For peer review only - http://bmjopen.bmj.com/site/about/guidelines.xhtml

being under-immunised for tetanus and MMR might benefit from a “catch-up” campaign, and the PED would seem to be a potential additional location for this to be delivered. Future work will also look to co-design one or more interventions to support MMR catch-up in this age group, as well as exploring the potential for the development of other vaccination-focused programmes that could be delivered in the case of an outbreak e.g. mumps, or for routine vaccination e.g. influenza.

Having identified this under-immunised population attending the PED, it may be that there are other under-immunised children and young people attending other settings within the hospital who might benefit from the offer of a vaccination-focussed intervention. Identification of these populations e.g. those attending outpatient clinics, would facilitate more opportunities to offer vaccination interventions as part of routine hospital care.

Funding statement

This work received no direct funding, but RI was supported by a Health Education England Topol Digital Health Fellowship from February 2021 for a year.

Competing interests statement

None declared.

Author contributions

RI conceived of the study, led on the ethics application process, undertook the fieldwork, analysed the data, was involved in all stages of the preparation of this manuscript and is guarantor author. LB analysed the data and was involved in all stages of the preparation of this manuscript. FE supported data extraction and was involved in later drafts of the manuscript. RE supported data analysis and was

For peer review only - http://bmjopen.bmj.com/site/about/guidelines.xhtml

involved in later drafts of the manuscript. ND and JK were involved in oversight of the project and were involved in later drafts of the manuscript.

Data sharing agreement

Relevant data may be requested from the lead author and will be provided in anonymised format as soon as legally and ethically possible.

Ethics approval statement

Full prospective ethics approval was obtained from the North West – Greater Manchester East Ethics Committee (IRAS reference 278815, REC reference 20/NW/0423). Following an earlier part of the work, an ethics amendment was submitted and approved to move to “opt-out” consent for this part of the project (Amendment 1, substantial, non-CTIMP, approved 26 August 2021).

For peer review only

For peer review only - http://bmjopen.bmj.com/site/about/guidelines.xhtml

References

1. Plotkin S. History of vaccination. *Proc Natl Acad Sci*. 2014 Aug 26;111(34):12283–7.

2. Smallpox [Internet]. [cited 2022 Jul 1]. Available from: https://www.who.int/westernpacific/health-topics/smallpox

3. Watson OJ, Barnsley G, Toor J, Hogan AB, Winskill P, Ghani AC. Global impact of the first year of COVID-19 vaccination: a mathematical modelling study. *Lancet Infect Dis*. 2022 Jun 23 [cited 2022 Jul 1]. Available from: https://www.thelancet.com/journals/laninf/article/PIIS1473-3099(22)00320-6/fulltext

4. Childhood Vaccination Coverage Statistics - 2021-22. NHS Digital. [cited 2023 Jan 13]. Available from: Childhood Vaccination Coverage Statistics- England, 2021-22 - NDRS (digital.nhs.uk)

5. Vaccination coverage for the second dose of measles-containing vaccine, EU/EEA, 2018 [Internet]. European Centre for Disease Prevention and Control. 2021 [cited 2022 Jul 1]. Available from: https://www.ecdc.europa.eu/en/publications-data/vaccination-coverage-second-dose-measles-containing-vaccine-eueea-2018

6. Hospital Episode Statistics » A&E Attendances and Emergency Admissions. [cited 2022 Jul 1]. Available from: https://www.england.nhs.uk/statistics/statistical-work-areas/ae-waiting-times-and-activity/

7. Isba R, Edge R, Jenner R, Broughton E, Francis N, Butler J. Where have all the children gone? Decreases in paediatric emergency department attendances at the start of the COVID-19 pandemic of 2020. *Arch Dis Child.* 2020 Jul 1;105(7):704–704.

8. Isba R, Edge R, Auerbach M, Cicero MX, Jenner R, Setzer E, et al. COVID-19. *Pediatr Emerg Care.* 2020. 36; 11: 551-553 [cited 2022 Jul 1]; Available from: https://www.ncbi.nlm.nih.gov/pmc/articles/PMC7493767/

9. Jenner R, Walker A, Isba R. Kids are back in town: the return of high demand for paediatric emergency care. *Arch Dis Child.* 2022 Feb 1;107(2):204–5.

10. Isba R, Edge R. Delivery of a multi-focus public health intervention in the paediatric emergency department: a feasibility and acceptability pilot study. *BMJ Open*. 2021 Dec 1;11(12):e047139.

11. NHS Digital. Summary Care Records (SCR). [cited 2023 May 4]. Available from: https://digital.nhs.uk/services/summary-care-records-scr

12. Newell K, Rousseva C, Slade C, Isba R. Should we offer opportunistic vaccination in the paediatric emergency department? *Emergency Medicine Journal* 2015; 32: 1007-1008

For peer review only

For peer review only - http://bmjopen.bmj.com/site/about/guidelines.xhtml

13. UK Parliament. UK Vaccination Policy. *Houses of Commons Library.* 2022. Available from: https://commonslibrary.parliament.uk/research-briefings/cbp-9076/

14. Public Health England. National Immunisation Programme: health equity audit. February 2021.

15. Bladgen S, Newell K, Ghazarians N, Odunala M, Sulaiman S, Tunn L, et al. Interventions delivered in secondary or tertiary medical care settings to improve routine vaccination uptake in children and young people: a scoping review. *BMJ Open*. Accepted with minor revisions.

16. Cunningham SJ. Providing immunizations in a pediatric emergency department: underimmunization rates and parental acceptance. *Pediatr Emerg Care.* 1999;15(4):255–9.

17. Bell LM, Pritchard M, Anderko R, Levenson R. A Program to Immunize Hospitalized Preschool-aged Children: Evaluation and Impact. *Pediatrics*. 1997 Aug 1;100(2):192–6.

18. Tarca AJ, Lau GT, Mascaro F, Clifford P, Campbell AJ, Taylor E. Pre- and post-intervention study examining immunisation rates, documentation, catch-up delivery and the impact of a dedicated immunisation service at a tertiary paediatric hospital. *J Paediatr Child Health*. 2021;57(2):263–7.

19. Hutchison RL, O'Rear J, Olson-Burgess C, Myers AL. Offering the Influenza Vaccine in a Pediatric Hand Surgery Clinic Increases Vaccination Rates. *J Hand Surg*. 2018 Aug 1;43(8):776.e1-776.e4.

20. Cameron MA, Bigos D, Festa C, Topol H, Rhee KE. Missed Opportunity: Why Parents Refuse Influenza Vaccination for Their Hospitalized Children. *Hosp Pediatr.* 2016 Sep 1;6(9):507–12.

21. Child and Maternal Health - PHE. [cited 2021 Feb 6]. Available from: https://fingertips.phe.org.uk/profile/child-health-profiles/data#page/0/gid/1938133228/pat/6/par/E12000002/ati/202/are/E08000003/iid/93700/age/169/sex/4/cid/4/tbm/1/page-options/ovw-do-0

22. GOV.UK Complete routine immunisation schedule. [cited 2022 Jul 1]. Available from: https://www.gov.uk/government/publications/the-complete-routine-immunisation-schedule

23. Isba R. Should we use Summary Care Records to access vaccination data in the Paediatric Emergency Department? Child Health Technology Conference. 2022.

24. Childhood Vaccination Coverage Statistics - England 2018-19. NHS Digital. [cited 2022 Jul 1]. Available from: https://digital.nhs.uk/data-and-information/publications/statistical/nhs-immunisation-statistics/england-2018-19

25. Stata: Software for Statistics and Data Science. [cited 2022 Jul 1]. Available from: https://www.stata-uk.com/software/stata.html/?utm_medium=adwords&utm_campaign=&utm_sour

For peer review only - http://bmjopen.bmj.com/site/about/guidelines.xhtml

ce=&gclid=Cj0KCQjwtvqVBhCVARIsAFUxcRvI4U2isNRQO_OqwQ6soXemgNuKF82amyEUsbw5v7cmOq2Hwhtvx4caApvAEALw_wcB

26. NHS Digital. Appendices - coverage definitions. [cited 2022 Jul 1]. Available from: https://digital.nhs.uk/data-and-information/publications/statistical/nhs-immunisation-statistics/england---2020-21/appendices#appendix-b-coverage-definitions

27. World Health Organization. Health21: the health for all policy framework for the WHO European Region. Copenhagen: World Health Organization, Regional Office for Europe; 1999. 224 p. (European health for all series).

Figure Legend:

Figure 1 – Level of vaccination coverage by age

For peer review only

Figure 1 - Level of vaccination coverage by age

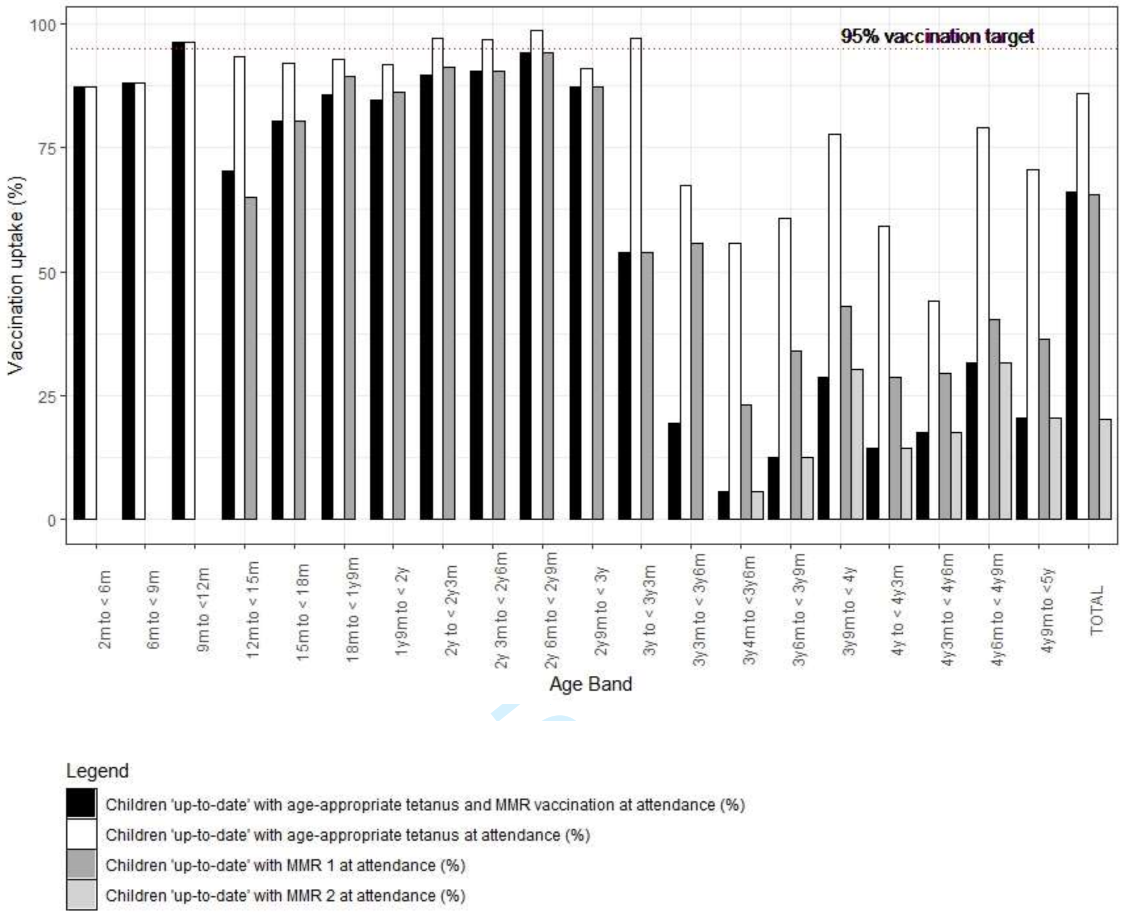

*Data to support figure 1 is included in supplementary file 1

Supplementary File 1 – Age distribution of children in the study

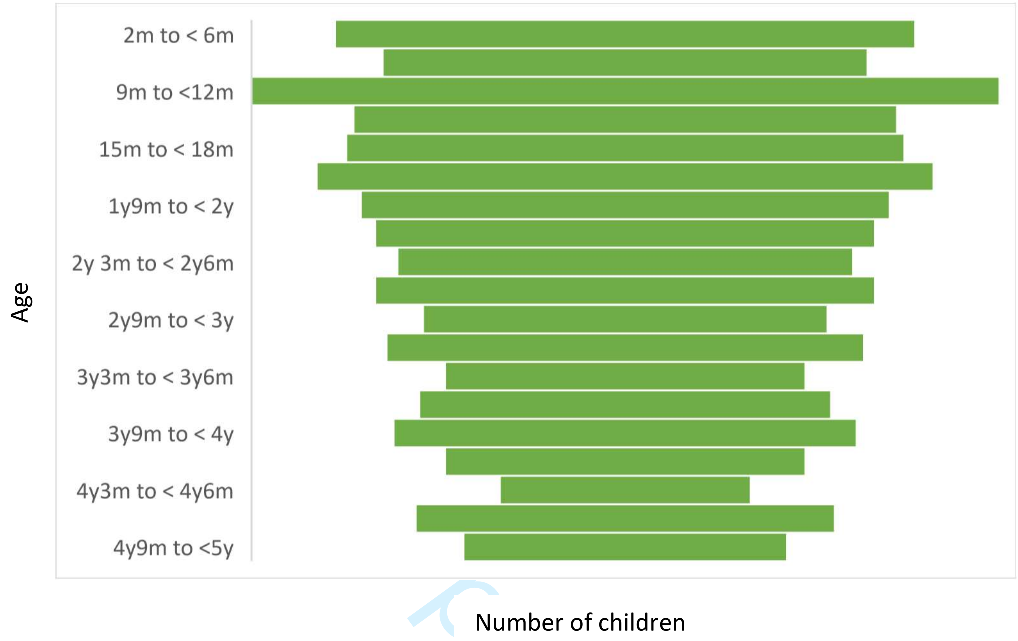

For peer review only

Supplementary file 2 – Data to support figure 1

Age band	Total number of children attending	Total number of children attending with vaccination data available	Children 'up-to-date' with age-appropriate tetanus and/or MMR vaccination at attendance (n (%))	Children 'up-to-date' with age-appropriate tetanus at attendance (n (%))	Children 'up-to-date with MMR 1 at attendance (n (%))	Up to date with MMR 2 at attendance
2m to < 6m	91	79	69 (87.3)	69 (87.3)		
6m to < 9m	74	66	58 (87.9)	58 (87.9)		
9m to <12m	113	102	98 (96.1)	98 (96.1)		
12m to < 15m	80	74	52 (70.3)	69 (93.2)	48 (64.9)	
15m to < 18m	86	76	61 (80.3)	70 (92.1)	61 (80.3)	
18m to < 1y9m	90	84	72 (85.7)	78 (92.9)	75 (89.3)	
1y9m to < 2y	77	72	61 (84.7)	66 (91.7)	62 (86.1)	
2y to < 2y3m	72	68	61 (89.7)	66 (97.1)	62 (91.2)	
2y 3m to < 2y6m	68	62	56 (90.3)	60 (96.8)	56 (90.3)	
2y 6m to < 2y9m	71	68	64 (94.1)	67 (98.5)	64 (94.1)	
2y9m to < 3y	62	55	48 (87.3)	50 (90.9)	48 (87.3)	
3y to < 3y3m	70	65	35 (53.8)	63 (96.9)	35 (53.8)	
3y3m to < 3y6m	53	49	7 (19.4)	33 (67.3)	20 (55.6)	
3y4m to <3y6m	*39*	*36*	*2 (5.6)**	*20 (55.6)*	*9 (23.1)*	*2 (5.6)*
3y6m to < 3y9m	60	56	7 (12.5)	34 (60.7)	19 (33.9)	7 (12.5)
3y9m to < 4y	69	63	18 (28.6)	49 (77.8)	27 (42.9)	19 (30.2)
4y to < 4y3m	53	49	7 (14.3)	29 (59.2)	14 (28.6)	7 (14.3)
4y3m to < 4y6m	39	34	6 (17.6)	15 (44.1)	10 (29.4)	6 (17.6)
4y6m to < 4y9m	59	57	18 (31.6)	45 (78.9)	23 (40.4)	18 (31.6)
4y9m to <5y	50	44	9 (20.5)	31 (70.5)	16 (36.4)	9 (20.5)
TOTAL	1337	1259	807 (66.0)	1050 (85.9)	640 (65.6)	68 (20.1)

*Denominator for calculation is number of children aged 3y4m < 3yr 6m (n=36)

For peer review only

Supplementary file 2 – Data to support figure 1

For peer review only

STROBE Statement—Checklist of items that should be included in reports of ***cross-sectional studies***

	Item No	Recommendation	Page No
**Title and abstract**	1	(*a*) Indicate the study's design with a commonly used term in the title or the abstract	1
		(*b*) Provide in the abstract an informative and balanced summary of what was done and what was found	2
**Introduction**			
Background/rationale	2	Explain the scientific background and rationale for the investigation being reported	4-8
Objectives	3	State specific objectives, including any prespecified hypotheses	8
**Methods**			
Study design	4	Present key elements of study design early in the paper	8
Setting	5	Describe the setting, locations, and relevant dates, including periods of recruitment, exposure, follow-up, and data collection	9
Participants	6	(*a*) Give the eligibility criteria, and the sources and methods of selection of participants	9
Variables	7	Clearly define all outcomes, exposures, predictors, potential confounders, and effect modifiers. Give diagnostic criteria, if applicable	11
Data sources/ measurement	8*	For each variable of interest, give sources of data and details of methods of assessment (measurement). Describe comparability of assessment methods if there is more than one group	9-10
Bias	9	Describe any efforts to address potential sources of bias	
Study size	10	Explain how the study size was arrived at	9
Quantitative variables	11	Explain how quantitative variables were handled in the analyses. If applicable, describe which groupings were chosen and why	10
Statistical methods	12	(*a*) Describe all statistical methods, including those used to control for confounding	12-13
		(*b*) Describe any methods used to examine subgroups and interactions	
		(*c*) Explain how missing data were addressed	
		(*d*) If applicable, describe analytical methods taking account of sampling strategy	
		(*e*) Describe any sensitivity analyses	
**Results**			
Participants	13*	(a) Report numbers of individuals at each stage of study—eg numbers potentially eligible, examined for eligibility, confirmed eligible, included in the study, completing follow-up, and analysed	13
		(b) Give reasons for non-participation at each stage	
		(c) Consider use of a flow diagram	
Descriptive data	14*	(a) Give characteristics of study participants (eg demographic, clinical, social) and information on exposures and potential confounders	13
		(b) Indicate number of participants with missing data for each variable of interest	13
Outcome data	15*	Report numbers of outcome events or summary measures	13
Main results	16	(*a*) Give unadjusted estimates and, if applicable, confounder-adjusted estimates and their precision (eg, 95% confidence interval). Make clear which confounders were adjusted for and why they were included	13-16

For peer review only

For peer review only - http://bmjopen.bmj.com/site/about/guidelines.xhtml

		(*b*) Report category boundaries when continuous variables were categorized	
		(*c*) If relevant, consider translating estimates of relative risk into absolute risk for a meaningful time period	
Other analyses	17	Report other analyses done—eg analyses of subgroups and interactions, and sensitivity analyses	
**Discussion**			
Key results	18	Summarise key results with reference to study objectives	16
Limitations	19	Discuss limitations of the study, taking into account sources of potential bias or imprecision. Discuss both direction and magnitude of any potential bias	18-19
Interpretation	20	Give a cautious overall interpretation of results considering objectives, limitations, multiplicity of analyses, results from similar studies, and other relevant evidence	19
Generalisability	21	Discuss the generalisability (external validity) of the study results	19
**Other information**			
Funding	22	Give the source of funding and the role of the funders for the present study and, if applicable, for the original study on which the present article is based	20

*Give information separately for exposed and unexposed groups.

Note: An Explanation and Elaboration article discusses each checklist item and gives methodological background and published examples of transparent reporting. The STROBE checklist is best used in conjunction with this article (freely available on the Web sites of PLoS Medicine at http://www.plosmedicine.org/, Annals of Internal Medicine at http://www.annals.org/, and Epidemiology at http://www.epidem.com/). Information on the STROBE Initiative is available at www.strobe-statement.org.

For peer review only
